# Supplementary material for: A Potential Biomarker of Dynamic Change in Peripheral CD45RA−CD27+CD127+ Central Memory T Cells for Anti-PD-1 Therapy in Patients with Esophageal Squamous Cell Carcinoma
Source: Cancers (Basel). 2023 Jul 16;15(14):3641. doi: 10.3390/cancers15143641 (PMC10377516; doi:10.3390/cancers15143641)

Supplementary Figure S1

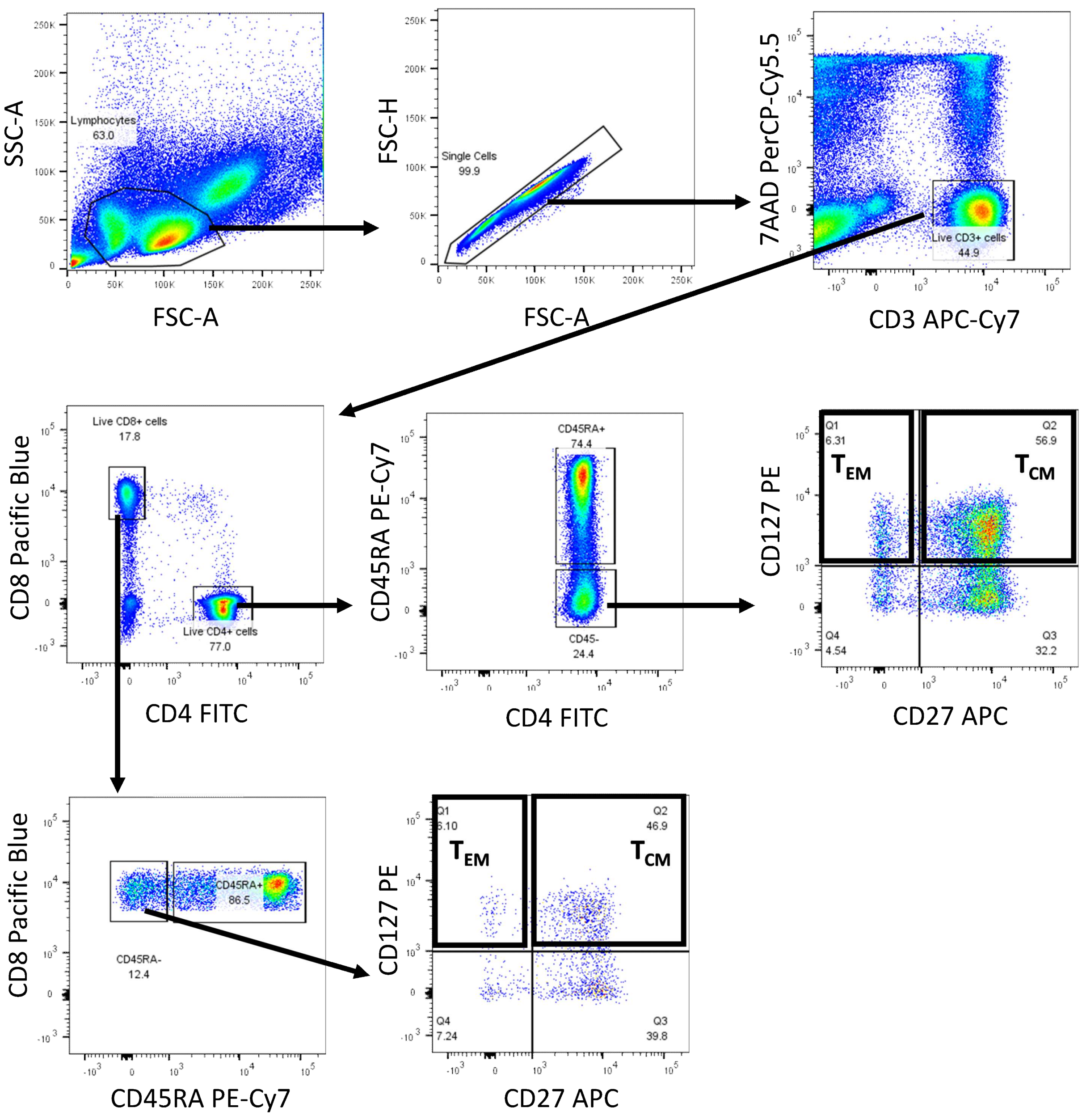

Supplementary Figure S2

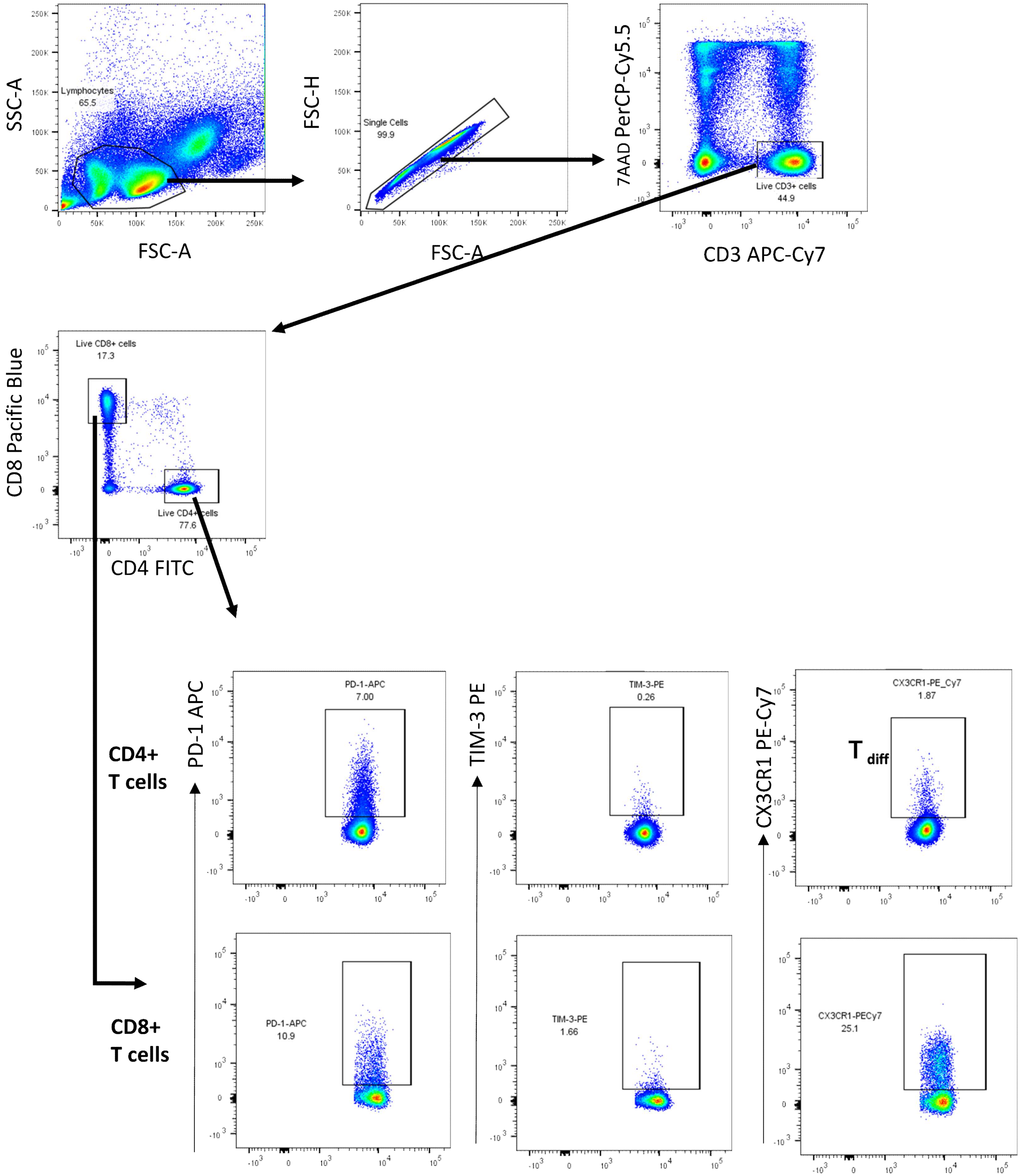

Supplementary Figure S3

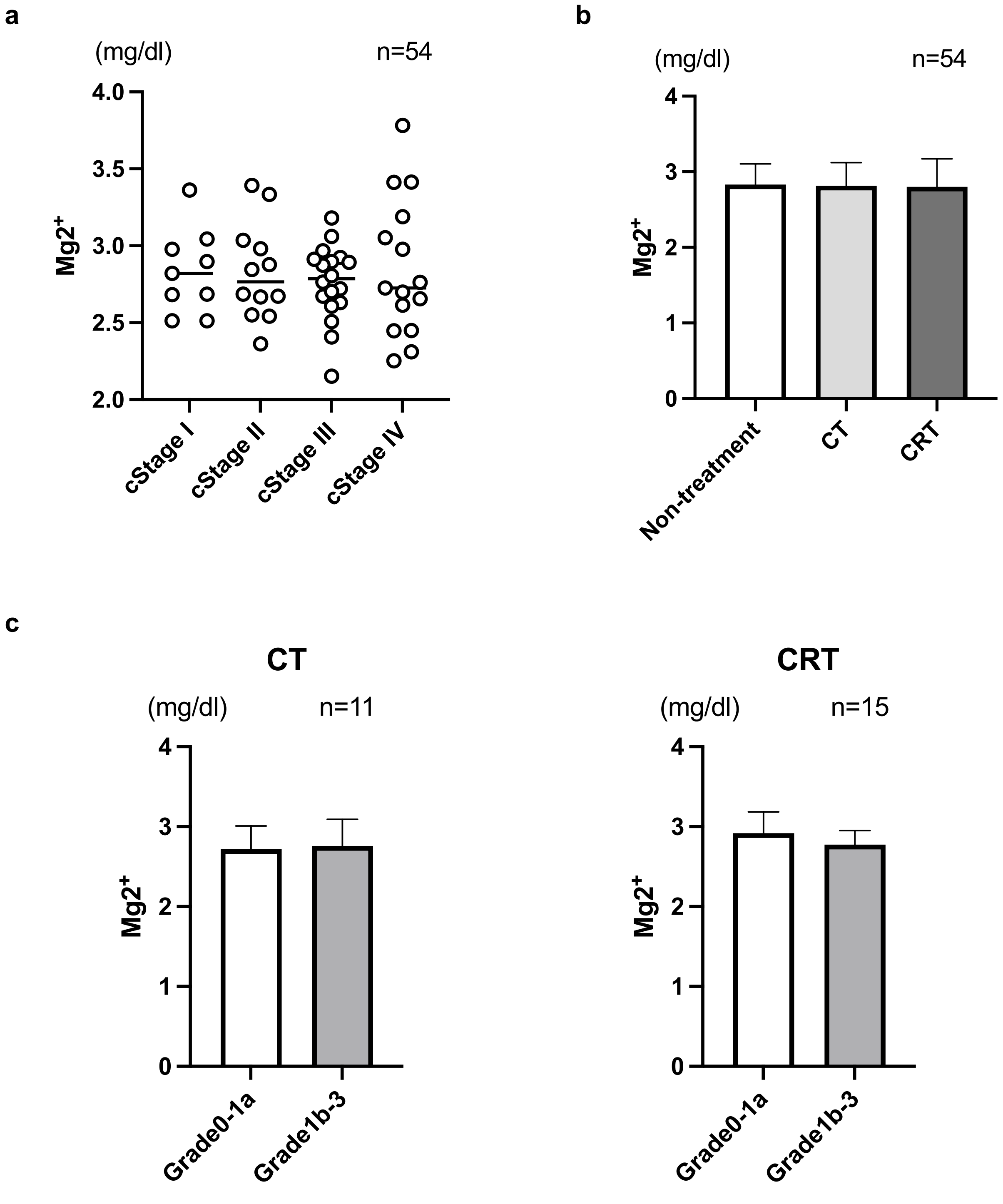

Suplementary Figure S4

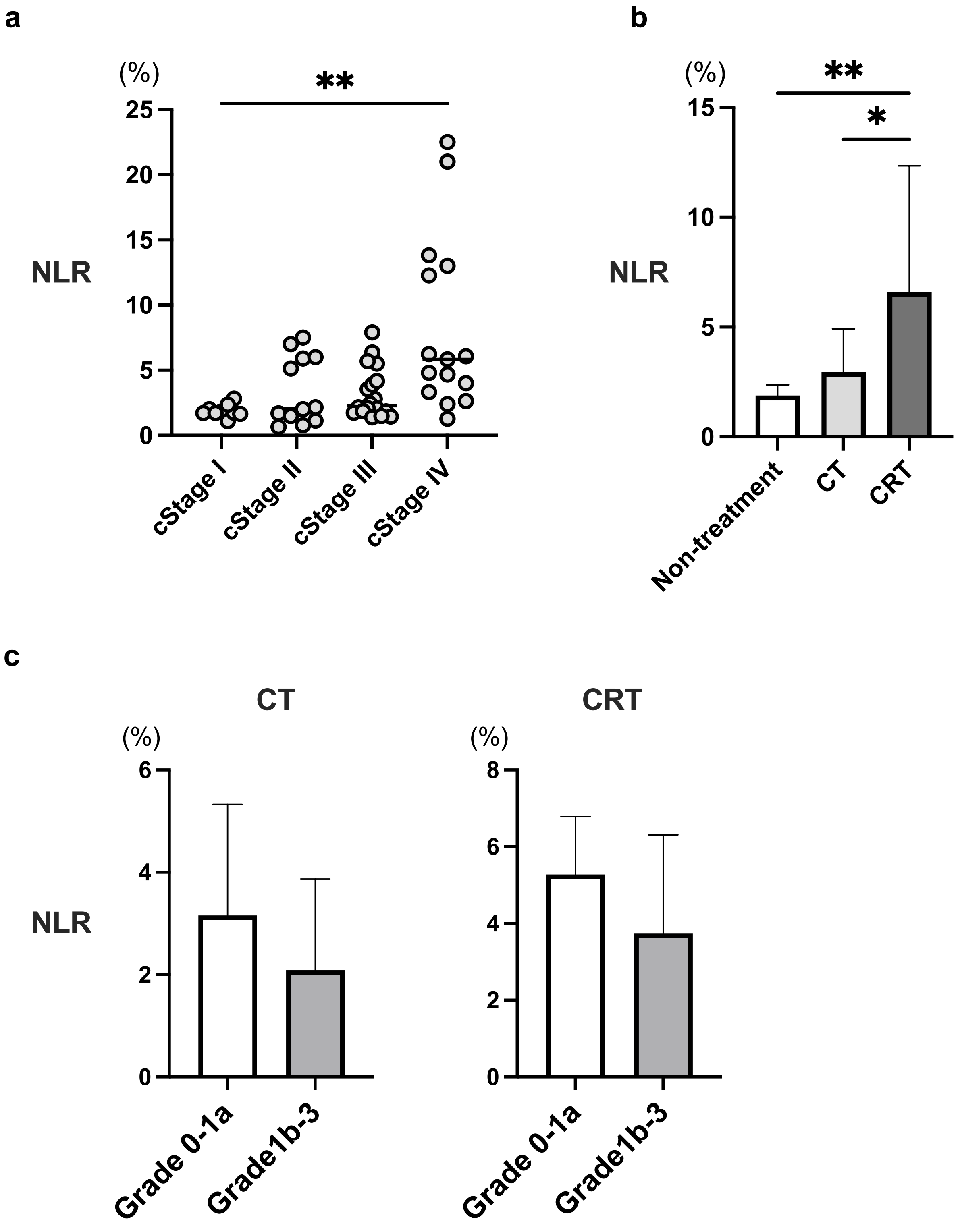

Supplement: Supplementary file 1 [file cancers-15-03641-s001.zip › cancers-2414938-supplementary.pdf]
